# Corruption, Hidden Economy and Environmental Pollution: A Spatial Econometric Analysis Based on China’s Provincial Panel Data

**DOI:** 10.3390/ijerph16162871

**Published:** 2019-08-11

**Authors:** Shi Wang, Yizhou Yuan, Hua Wang

**Affiliations:** 1 School of Economics and Finance, Xi’an International Studies University, Xi’an 710128, China; 2 Institute of International Public Opinion and International Communication, Xi’an International Studies University, Xi’an 710128, China; 3 “One Belt One Road” Economic and Trade Cooperation Innovation Team, Xi’an International Studies University, Xi’an 710128, China; 4 School of Foreign Studies, Xi’an Jiaotong University, Xi’an 710049, China

**Keywords:** environmental pollution, corruption, hidden economy, spatial econometric analysis

## Abstract

Previous studies show that the environmental quality is significantly influenced by corruption and the hidden economy separately. However, what is the impact of their interaction effect on environmental quality? Based on Multiple Indicators Multiple Causes (MIMIC) model, this study calculates the scale of hidden economy in Chinese provinces firstly. Then, we apply the method of spatial econometrics to analyze the interaction effect of corruption and the hidden economy on environmental pollution with China’s provincial panel data from 1998 to 2017. The results indicate that the interaction effect between corruption and hidden economy significantly increases pollutant discharge, suggesting that both anti-corruption and control of the hidden economy may improve environmental quality directly and indirectly.

## 1. Introduction

Although China has accomplished remarkable achievements in economic development since the reform and opening-up, the consequent corruption has become a major challenge for Beijing. Transparency International’s annual Corruption Perceptions Index (CPI) discloses that China has consistently been ranked around 80th in the last five years, the worst rank being 100th in 2014, indicating the prevalence and seriousness of the corruption challenge in China. Corruption not only impedes economic growth but also affects environmental policies and environmental quality. Corruption at the level of the central government may result in suspension or delay of environmental policies or reduction in policy intensity. Corruption at the local government level may block the enforcement of environmental policies in enterprises and result in the decline of the standards of policies. As a result, high-polluting enterprises that used to be forbidden will enter the market and start to organize production, and enterprises using cleaner production technologies will stop the application of emission-control devices or switch to polluting production technologies to save cost, thus increasing the pollutant discharge.

Another potentially unfavorable factor accompanying China’s rapid economic growth is the expansion of the hidden economy, also known as shadow economy, underground economy, or unofficial economy [1,2]. It is most commonly defined as “all economic activities that are not officially counted” [3]. Not being regulated by the government, the hidden economy has caused many economic and social issues including environmental problems. Blackman [4] found that hidden economic activities can significantly harm the environmental quality because they involve many pollution-intensive production activities which fail to meet the requirements of environmental regulations.

Numerous studies have investigated the effects of corruption or hidden economy on environmental pollution, as Section 2 elaborates, but the impact of their interaction effect has been neglected. Intuitively, the stricter the government regulation is, the less likely the enterprise is to engage in hidden production activities. However, corruption will weaken the intensity of government regulation, thus expanding the size of the hidden economy and increasing the volume of pollutants discharged. Based on China’s provincial panel data, this study conducts an empirical analysis about the influence of the interaction effect between corruption and the hidden economy on environmental quality.

## 2. Literature Review

### 2.1. Corruption and Environmental Pollution

Corruption can directly affect environmental regulations and environmental pollution. In the case study concerning developing countries in Southeast Asia conducted by Desai [5], it is found that polluting enterprises, by bribing government officials, could influence or delay the legislative process of environmental laws and reduce the enforcement intensity of existing environmental regulations, thus giving rise to elevated pollutant discharge. Pellegrini and Gerlagh [6] carried out an empirical study by employing the panel data of 22 European countries and found that corruption imposes a negative effect on environmental regulations. They also found that corruption is an important source of differences in environmental regulations among European countries, even more than the impact of economic differences. Based on the panel data of 39 countries from 1999 to 2003, Ivanova [7] found that polluting enterprises in countries with serious corruption problems tended to underreport their pollutant discharge to evade environmental taxes. Empirical studies by Hubbard [8] and Oliva [9] also indicate that corruption leads to the decrease in the effectiveness of the policies targeting at vehicle emission control. Using the quantile regression approach, Zhang et al. [10] developed a panel data model for the effect of corruption on CO_2_ emissions in APEC countries. The empirical results show that there is a significant negative effect in lower emission countries, but the effect becomes non-significant in higher emission countries. The study by Lisciandra and Migliardo [11] reveals that corruption is harmful for environmental quality in general based on the comprehensive environmental quality indicator (EPI) and panel VAR analysis. Using the panel data of 87 countries from 2000 to 2012, Chang and Hao [12] analyzed the relationship between environmental performance, corruption and economic growth with static and dynamic panel models. They measured environmental quality with the Environmental Performance Index and found that lower corruption will weaken the positive relationship between economic growth and environmental performance. Lapatinas et al. [13] found that in the presence of corruption, the implementation of technologically advanced environmental policies may result in lower environmental quality. With a partial least square regression model and panel data of BRICS economies from 1996 to 2015, Wang et al. [14] analyzed the moderating role of corruption in the relationship between growth and CO_2_ emissions, and found lower corruption reduces CO_2_ emissions. Combined with a new index of corruption in each state of the US, Dince and Fredriksson [15] found that higher corruption weakens environmental regulation and improves the pollutant discharge when the level of trust is low. Using the simultaneous equations framework and data from 1985 to 2011, Arminen and Menegaki [16] found that the effect of corruption on energy consumption and CO_2_ emissions is very limited and much weaker than climate and weather variations. With a large cross-country data set, DiRienzo and Das [17] found that female officials reduce environmental pollution by the indirect effects of reducing corruption. Using panel data from 16 countries during 1990 to 2017, Sinha et al. [18] found that corruption worsens the environmental quality by reducing the positive effect of renewable energy consumption on environmental quality and increasing the negative effect of fossil fuel consumption. Their study also shows that corruption is more likely to occur in countries with stricter regulation. Other related studies are the work by Balsalobre-Lorente et al. [19] and Huynh and Hoang [20].

### 2.2. Hidden Economy and Environmental Pollution

Several papers explore the relationship between the hidden economy and environmental pollution. Many scholars find that the hidden economy is harmful for the environmental quality. Chaudhuri and Mukhopadhyay [21] probed into the effectiveness of environmental regulations on hidden economic sectors. The results suggest that when environmental regulations are stricter, formal production activities are more likely conducted in a hidden way. Therefore, if a government does not impose any restrictions on hidden economic sectors, the effects of environmental regulations are greatly reduced. By building a theoretical model, Baksi and Bose [22] analyzed the effects of environmental regulations on the existence of a hidden economy. It was found that the rise of the level of environmental regulations forces formal departments to transfer their economic production activities into hidden sectors through a “structure effect”, hence increasing the pollutant discharge. The study by Elgin and Mazhar [23] indicates that the nominal level of government regulations, the enforcement intensity of environmental regulations and the policy quality are important factors of environmental pollution, and that the hidden economy can intensify environmental pollution by weakening the enforcement intensity of environmental regulations. In addition, an increase of environmental regulations will not necessarily reduce pollution since it will push more economic activities in formal sectors to be transferred into the hidden economy. The pollution can only be reduced if the enforcement intensity of policies is ensured. Based on the Environmental Kuznets Curve framework and Tunisia’s data from 1980 to 2009, Abid [24] found that the expansion of the hidden economy increases environmental pollution. On the basis of the research conclusion, the author puts forward policy suggestions to reduce the scale of the hidden economy and carbon dioxide emission without affecting economic growth. By using panel data from 30 provinces of China during the period from 1998 to 2012 and employing the generalized method of moments (GMM) to control potential endogeneity and introduce dynamic effects, Chen et al. [1] found that stringent environmental regulation and the level of the shadow economy are both positively related to China’s environmental pollution. Imamoglu [25] used data of Turkey from 1970 to 2014 and time series analysis to examine the relationship between the scale of hidden economic activity and environmental quality. The results show that both formal and hidden economy have great impact on environmental quality. However, compared with the hidden economy, the formal economy has a greater impact on environmental quality. The Turkish authority needs to focus more on formal and hidden economic activities to prevent environmental degradation in Turkey. Canh et al. [26] analyzed the effects of income level, hidden economy, urbanization, industrialization, energy intensity, public expenditure, trade openness and FDI inflow on the emission of carbon dioxide, CH_4_ and other pollutants with panel data of 106 economies from 1995 to 2012 and STIRPAT model. They found that industrial energy intensity is the main driver of all emissions. The hidden economy increases all emissions except carbon dioxide. Based on panel data from 30 Chinese provinces during 1998 to 2016, Zhou [27] found an inversely N-shaped relationship between carbon dioxide emissions and income level in China’s provinces, even when the hidden economy is taken into account.

Some scholars found evidence of a nonlinear relationship between the hidden economy and environmental pollution. Using the panel data of 152 countries from 1999 to 2009, Elgin and Oztunali [28] found that there is an inverted U-shaped relationship between them. More specifically, the increase of the hidden economy’s size promotes pollutant discharge when the size of the hidden economy is relatively small, whereas the hidden economy’s effects are reversed if the size of the hidden economy exceeds the critical value. Furthermore, the authors explored the reason behind such relationship by constructing a dynamic general equilibrium model. The results suggest that the hidden economy affects environmental pollution through two mechanisms, the size effect and the deregulation effect. The former suggests that, compared with enterprises of formal sectors, enterprises of hidden sectors are usually not capital-intensive and have a relatively small production scale. Hence pollution caused by individual enterprises of this kind is not so serious compared with the pollution caused by enterprises in the formal sectors. The latter implies that one of the essential characteristics of hidden economy is that the hidden economy is not subject to government regulations. Although the hidden economy alleviates environmental pollution through the former mechanism, it intensifies pollution through the latter one. Given that the two mechanisms work in opposite directions, the relationship between them takes the shape of an inverted U.

### 2.3. Limitations of Existing Research

While the research on the influence of corruption or hidden economy on environmental quality is extensive, it pays little attention to the impact of their interaction effect. To our knowledge, Biswas et al. [29] are the only researchers that have investigated this question. In our opinion, their research can further be extended at least in the following two aspects. Firstly, in terms of the measurement of corruption level, Biswas et al. [29] used the corruption index issued by Political Risk Services Inc. (PRS) and CPI by Transparency International, both of which measure the annual corruption level only at the national level, thus neglecting corruption at the regional level. Secondly, they adopted the traditional method of panel data regression to conduct the empirical analysis based on the underlying assumption that pollutant discharges of different regions do not affect each other. In fact, there is a spatial correlation of environmental quality due to natural factors like wind direction and water flow as well as economic ties between different regions. In other words, the environmental quality of one region can be influenced by that of other regions and the spatial correlation of environmental quality will be further enhanced by regional corruption and externalities of environmental policies. If the spatial co-movement is ignored during the empirical analysis process, the results obtained may be biased [30]. Therefore, it is necessary to use the spatial panel econometric method for an in-depth and accurate research.

## 3. Calculation of the Size of the Hidden Economy in China’s Provinces

This section will calculate the size of the hidden economy in China’s provinces. Firstly, we briefly introduce common measure methods of the hidden economy used in the literature. Secondly, the method chosen by this study, Multiple Indicators Multiple Causes (MIMIC) model, is described in detail, along with all possible cause and indicator variables involved in the model. Thirdly, MIMIC model is estimated. Finally, the scale of hidden economy in Chinese provinces from 1998 to 2017 is calculated.

### 3.1. Calculation Method of the Hidden Economy

The size of the hidden economy can be calculated either directly or indirectly. Direct calculation can be achieved by microeconomic approaches like actual surveys and tax audit. Actual surveys are widely used to calculate the size of the hidden economy in many previous studies, such as Cantekin and Elgin [31] for Turkey and Medvedev and Oviedo [32] for Ecuador. Tax audit, which is designed to measure the amount of undeclared taxable income, can be used to calculate the size of the hidden economy as well [33]. Although the direct calculation approaches mentioned above can provide details about hidden economy, they lead only to point estimates and cannot capture development and growth of the hidden economy for all provinces in China over a long period of time [34]. As a result, the direct calculation approaches cannot be used in this study. Indirect calculation, also termed as indicator calculation, is to indirectly measure the size of the hidden economy by applying various macroeconomic indicators containing hidden economic information, such as transaction [3], currency demand [35], and Multiple Indicators Multiple Causes (MIMIC) model, of which the last two are the most commonly used. Orsi et al. [36] provide a detailed discussion of the advantages and disadvantages of these indirect calculation methods. The MIMIC model considers multiple causes and effects of the hidden economy simultaneously, in contrast to other approaches that only consider one indicator [34]. So it can measure the size of the hidden economy more accurately, and is widely used in the related literature, such as Bajada and Schneider [37] for Asia-Pacific countries, Dell’Anno and Solomon [38] for the United States and Popescu et al. [39] for Romania. In addition, currency data at the provincial level are not available in China. Consequently, this study uses the MIMIC model to calculate the size of the hidden economy in different provinces of China. Following the research by Giles [40], MIMIC model structurally consists of two parts including the measurement model for the relationship between indicator variables and unobservable variables (the size of the hidden economy in this study) and the structural model for the relationship between the hidden economy and causal variables. The measurement model can be expressed as:

(1) $$Y_{1}=\lambda_{1}\times \eta +\varepsilon_{1},Y_{2}=\lambda_{2}\times \eta +\varepsilon_{2},\cdots ,Y_{p}=\lambda_{p}\times \eta +\varepsilon_{p}$$

$Y_{1}$,$Y_{2}$,$\cdots ,Y_{p}$ represent a set of observable indicator variables related to the hidden economy, and η represents hidden economy. $\lambda_{1}$,$\lambda_{2}$,⋯,$\lambda_{p}$ represent parameters of measurement model, and ε represents measurement error.

The structural model can be expressed as:

(2) $$\eta =\gamma_{1}\times Z_{1}+\gamma_{2}\times Z_{2}+\cdots +\gamma_{k}\times Z_{k}+\xi$$

$Z_{1}$,$Z_{2}$,⋯,$Z_{k}$ represent a set of observable cause variables, and η represents hidden economy. $\gamma_{1}$,$\gamma_{2}$,⋯,$\gamma_{k}$ represent parameters of structural model, and ξ represents random disturbance term.

Equations (1) and (2) above can be rewritten as vector form:

(3) $$\vec{Y}=\vec{\lambda }\times \eta +\vec{\varepsilon }$$

(4) $$\eta =\vec{\gamma }\times \vec{Z}+\xi$$

In order to solve the model, we substitute the Equation (4) into the Equation (3). The MIMIC model can be expressed in the form of the following multiple regression equation:

(5) $$\vec{Y}=\vec{\lambda }\times (\vec{\gamma }\times \vec{Z}+\xi )+\vec{\varepsilon }$$

$\vec{\lambda }$ and $\vec{\gamma }$ represent parameter vector.

**Figure 1:**
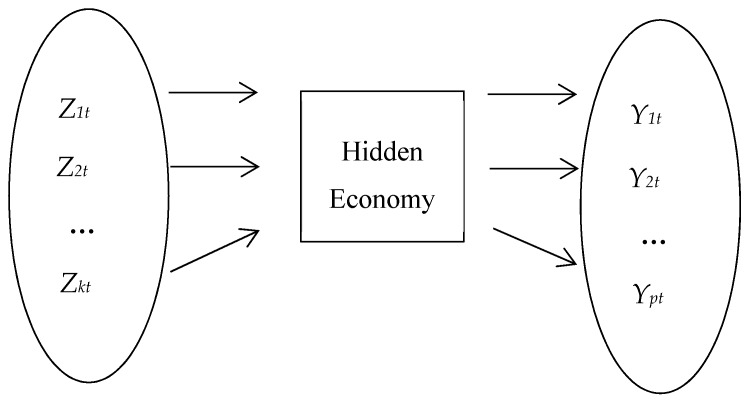
A Multiple Indicators Multiple Causes model.

Before evaluating Equation (5), we need to preset a value for an element in the vector $\vec{\lambda }$ and then normalize it. In other words, the estimation of MIMIC model needs to construct a scale index. Following Alanon and Gomez-Antonio [41], output index is usually taken as the scale index. If the model is correct and can be identified, the parameter vectors $\vec{\lambda }$ and $\vec{\gamma }$ can be obtained by using the maximum likelihood ratio method. Suppose the average value of the random disturbance term ξ is 0, then the ordinal value of the hidden economy η, or hidden economy index(SE*) as shown below, can be calculated using Equation (4). However, the ordinal value obtained must be converted to the absolute value, which is the share of the hidden economy in GDP, represented by SE below. So it is important to get the absolute value of a sample point. The common practice is to use other estimation methods (such as the money demand method, etc.) to obtain the absolute value. MIMIC model mentioned above can also be shown in Figure 1.

In the figure, Z_it_ (i = 1,2,...,k)are causes concerning the hidden economy, while Y_jt_ (j = 1,2,…,p)are indicators.

### 3.2. Explanation of Causes and Indicators

Thomas [42] points out that when using MIMIC model, the causes and indicators must be selected appropriately. Due to specific national conditions, the reasons and characteristics of hidden economy in different countries not only have certain commonness but also show some unique features. Therefore, the selection of causes and indicators is different for research in different countries. In most related studies, the tax burden, resident income, unemployment rate, government regulation and self-employment rate are selected as causes or explanatory variables in Equations (2) and (4), and economic growth, labor force participation rate and currency in circulation are selected as indicators or dependent variables in Equations (1) and (3) [37,40,43]. When calculating the size of hidden economy in China’s provinces with MIMIC model, we select as many variables used in the literature as possible to ensure the accuracy of the measurement and make some adjustments based on the actual situation of the country. For example, China does not publish the data of currency in circulation in provinces, so this study does not take currency in circulation as an indicator variable. The details of causes and indicators we finally select follow next.

#### 3.2.1. Causes

Tax burden (TT). Tax burden is the most important determinant of the hidden economy. Schneider [44] and Schneider and Dell’Anno [45] found that the impact of tax burden on the hidden economy is very significant. Research by Schneider [46] on three Nordic countries (Denmark, Norway and Sweden) revealed similar results, that is, average direct tax rate, average total tax rate (including direct tax and indirect tax) and marginal tax rate all have a significant positive impact on the hidden economy. Specifically, the increase of tax burden will increase the incentive for enterprises to transfer production activities from the formal sector to the informal sector.

Tax burden affects the choice of workers as well. If there is a large gap between the total living cost and after-tax income in the formal economy, workers will have a strong incentive to work in the hidden economy in order to make up the gap by avoiding taxes [44,45]. In this study, the tax burden is expressed as the share of provincial tax revenue in GDP. In addition, the total tax is divided into direct tax and indirect tax to analyze the impact of different kinds of taxes on the hidden economy, and the direct tax burden (DT) and indirect tax burden (IT) are calculated separately.

Resident income (INC). In the national income distribution (including the primary distribution and redistribution), the lower the proportion of resident sector distribution, the stronger the willingness of individual residents to increase their personal income by participating in hidden economic activities and the larger the hidden economy scale.

Unemployment rate (UNE). In the labor market, the formal economy can provide jobs with stability and social security, while the jobs in the hidden economy tend to be of short term and high mobility. The hidden economy does not need to provide workers with social security and welfare payments in addition to their wages, and its employment costs are lower than that in the formal economy.

Many studies have shown that excessive regulation of the labor market (such as restrictions on the working hours per week, early retirement, etc.) and high employment costs will result in the labor force shifting from the formal economy to the hidden economy [47]. Saafi and Farhat [48] found that the unemployed have more time to work in the hidden economy. Bajada and Schneider [37] found that the reemployment rate of the hidden economy is much higher than that of the formal economy, and the unemployed in the formal economy are more likely to find jobs in the hidden economy.

The rising of unemployment rate, on one hand, increases the idle labor force in the labor market and provides sufficient labor supply for the hidden economy; on the other hand, it brings down the cost of labor and provides cheaper labor for the hidden economy. In conclusion, unemployment rate is an important factor affecting the size of the hidden economy, and the higher the unemployment rate of the formal economy is, the more people who are unemployed engage in the hidden economy for the sake of living. Since China only published the urban registered unemployment rate, this study will use this index to measure the unemployment rate.

Government regulation (GR). Schneider and Enste [47] found that government regulation is an important factor affecting hidden economy. Ihrig and Moe [49] found a positive correlation between government regulation and the hidden economy. However, this study argues that there may be a more complicated relationship between government regulation and hidden economy. On one hand, excessive regulation (such as permit system and trade barriers) raises the entry costs; the low efficiency of public services and tedious approval procedures raise the transaction costs for investors. As a result, enterprises and individuals that want to enter the formal economy turn to the informal sector. On the other hand, if the government strengthens supervision and improves laws and regulations, it can avoid corruption and deter tax evasion. Consequently, the size of the hidden economy will be reduced. Government regulation is expressed in terms of the proportion of public servants in the total employed population.

Self-employment rate (SFE). The ratio of self-employment in total labor is one of the important factors affecting the hidden economy. Bordignon and Zanardi [50] argue that self-employment has a great likelihood of evading taxes, leading to a reduction in the tax base and underreporting of personal income. This means that the higher the self-employment rate, the larger the hidden economy, and vice versa. Popescu et al. [39] and Schneider and Dell’Anno [45] found that the self-employment ratio is significantly positively correlated with the hidden economy. The hidden income increases the ratio of self-employed and private workers in the total labor.

From the results of the studies above, we find that the size of the hidden economy increases in the number of workers participating in the hidden economy. Since the reform and opening-up, China’s individual economy has been greatly developed, which not only promotes economic development and provides employment opportunities but also improves hidden economy. Therefore, the higher the self-employment rate is, the larger the hidden economy scale will be. In this study, the self-employment rate is expressed by the proportion of self-employment in urban and rural areas in the total employment.

#### 3.2.2. Indicators

Economic growth (RGDP). Schneider [44] finds that the relationship between the hidden economy and the formal economy in developed countries is pro-cyclical, while in developing countries it is counter-cyclical. Therefore, the influence of the hidden economy on the formal economy is uncertain. On one hand, the expansion of the hidden economy means the contraction of the formal economy when the total economy remains unchanged, so the hidden economy is negatively correlated with the formal economy. On the other hand, the hidden economy is not independent, and it is closely related to the formal economy. In the expansion phase of the economic cycle, with the increase of the official economic growth rate, the relative size of the hidden economy also increases because it meets the consumer demand for some goods and services that cannot be involved in the official economy [47]. For example, part of the hidden economic income is directly used for formal economic consumption, so the hidden economy promotes the development of the formal economy. This study measures economic growth in terms of real GDP per capita growth.

Labor force participation rate (LAB). An increase in the size of the hidden economy usually reflects a decline in the official labor force participation rate [39,43]. The larger the hidden economy is, the fewer workers will engage in the formal economy and the fewer hours workers will work in the formal economy. In this study, the labor force participation rate is expressed by the ratio of total employment to total population.

#### 3.2.3. Source of Data

Since China began to publish data on value-added tax and business taxes at the provincial level in 1998, the sample period is from 1998 to 2017. Data of total tax revenue, various indirect tax incomes, per capita disposable income of urban residents, per capita net income of farmers, GDP, total employment, urban and rural private and individual employment come from *China Statistical Yearbook*. Data of agricultural population and non-agricultural population come from *China Population & Employment Statistical Yearbook*. Data of registered urban unemployment rate and public servants come from *China Labor Statistical Yearbook.* Real GDP growth per capita is calculated using the per capita GDP index from *China Statistical Yearbook*.

### 3.3. Estimation Results of the MIMIC Model

MIMIC model in Equations (3) and (4) is a structural equation model, and we estimate with the maximum likelihood ratio method. The dependent variables in Equation (3) are indicators shown above, and the explanatory variable is hidden economy. The explanatory variables in Equation (4) are causes shown above, and the dependent variable is hidden economy. Following the studies by Biswas et al. [29], Schneider et al. [43] and Schneider [44], we start from the most general form of the model, which includes all indicators (economic growth, labor force participation rate) in Equation (3) and all causes (tax burden, direct tax burden, indirect tax burden, resident income, unemployment rate, government regulation, self-employment rate) in Equation (4). We stepwise exclude variables that are not statistically significant and determine the most suitable model according to the probability value of $\chi^{2}$, RMSEA, CFI and SRMR. We only present the estimated results of seven models, in which most estimated coefficients are statistically significant and consistent with the expectation, in Table 1.

According to the criterion of structural equation model, the bigger p value of $\chi^{2}$ and CFI value are, the better, and the smaller the value of RMSEA and SRMR are, the better. According to these criterions, model (7) in Table 1 is the optimal model. In addition, it can be found that all variables in model (7) are significant at least at the 5% level. Therefore, we will calculate the hidden economic scale based on model (7).

### 3.4. Calculation Results of the Hidden Economy and Analysis

As shown in Table 1, model (7) contains three causal variables, TT, UNE and SFE, and two indicator variables, RGDP and LAB. According to the estimation results, the following structural equation is generated:

(6) $$SE_{it}^{*}=0.12\times TT_{it}+1.11\times UNE_{it}-0.02\times SFE_{it}$$

In the equation, $SE_{it}^{*}$ is the index of the hidden economy, or the ordinal value of the hidden economy η mentioned above. By substituting the values of causal variables into Equation (1), the indexes of the hidden economy of different provinces from 1998 to 2017 can be obtained. It should be noted that this index needs to be converted into the hidden economy’s share of GDP. Specifically, one year is firstly determined as the base period, at which the hidden economy’s size is calculated by means of other estimation methods. In this study, the hidden economy’s share of GDP in 2000 is calculated using the elasticity coefficient estimation method. Subsequently, the hidden economy’s share of GDP in other years is calculated by employing the following equation:

(7) $$SE_{it}=SE_{i,2000}\times \frac{SE_{it}^{*}}{SE_{i,2000}^{*}}$$

In the equation, SE_it_ refers to the hidden economy’s share of GDP of province *i* in the year of *t*, or absolute value of the hidden economy η mentioned above, and SE_i,2000_ stands for the hidden economy’s share of GDP of all provinces in 2000 calculated by applying the method used by Li Jinchang and Xu Aiting [51]. By substituting the index of the hidden economy calculated by Equation (6) into Equation (7), the hidden economy’s shares of GDP from 1998 to 2017 can be calculated. The results are shown in Table A1 and Table A2 in the Appendix A, in which it is obvious that the hidden economy’s size during the sample period ranges from 10.7% to 14.2% on average, the eastern region ranging from 10.5% to 15.6%, the central region from 9.1% to 12.6% and the western region from 11.4% to 14.9%.

The results are similar to those of Li Jinchang and Xu Aiting [51]. In addition, we also found that the hidden economy’s size presents a trend of rising and then descending no matter whether in the whole country, the eastern, central or western regions. The average size of hidden economy of the whole country reached its peak in 2003, and gradually decreased hereafter, which indicates that the Chinese government has gradually strengthened its control over the hidden economy in recent years. The average size of hidden economy of the eastern region is similar to that of the whole country during the period of investigation. The western region is slightly higher than the national average level, whereas the central region has been lower than the national average level. This may be because provinces in western region have lower levels of governance than others.

## 4. Empirical Analysis of the Influence of Corruption and Hidden Economy on Environmental Pollution

### 4.1. Specifications of the Econometric Model

In this part, the conclusion of the theoretical model is empirically tested based on the panel data regression model. The following econometric equation is to estimate whether the relationship between hidden economy and environmental pollution is influenced by corruption level:

(8) $$EP_{it}=\beta_{0}+\beta_{1}COR_{it}+\beta_{2}SE_{it}+\beta_{3}COR_{it}\times SE_{it}+\beta_{4}Z_{it}+u_{i}+\varepsilon_{it}$$

In the equation, subscript *i* and subscript *t* represent province and time respectively. β_0_–β_4_ refers to parameters to be estimated, EP to the indicator of environmental pollution, COR to the proxy variable of corruption and SE to the indicator of the size of the hidden economy. Therefore, β_2_ measures the direct impact of the hidden economy on environmental pollution. COR*SE represents the interaction term of hidden economy and corruption, hence β_3_ suggests the extent to which corruption increases or decreases the impact of the hidden economy on environmental pollution. Z is the control variable that affects environmental pollution, u*_i_* the regional fixed effect which is invariant with time and ε*_it_* the random error term.

Tobler’s First Law of Geography claims that “everything is related to everything else, but near things are more related than distant things” [52]. Under the influence of natural factors, the environmental condition of a region is bound to be closely associated with the environmental quality of adjacent areas and the correlation of environmental quality is strengthened by the interrelation of economic production activities between different regions. In order to reflect the spatial correlation of environmental pollution, a Spatial Lag Model (SLM) and Spatial Errors Model (SEM) are constructed in this study.

Specifically, the spatial correlation is conceived to originate from explanatory variables, and it is only the pollutant discharge of adjacent areas that could affect the environmental quality of the local region in SLM. The corresponding econometric model is presented below:

(9) $$EP_{it}=\rho W\times EP_{it}+\beta_{0}+\beta_{1}COR_{it}+\beta_{2}SE_{it}+\beta_{3}COR_{it}\times SE_{it}+\beta_{4}Z_{it}+u_{i}+\varepsilon_{it}$$

ρ is the spatial regression coefficient, which describes the spatial correlation and reflects the direction and degree of the influence of pollutant discharge of adjacent areas on the environment quality of the local region. W is known as the N × N spatial weight matrix.

The spatial correlation is represented by the error term. This term explains missing variables exclusive to the known explanatory variables that are spatially correlated and can influence local environmental quality or unpredictable random shocks with spatial correlation. The corresponding econometric model is shown below:

(10) $$\begin{array}{c} EP_{it}=\beta_{0}+\beta_{1}COR_{it}+\beta_{2}SE_{it}+\beta_{3}COR_{it}\times SE_{it}+\beta_{4}Z_{it}+u_{i}+\varepsilon_{it}\\ \varepsilon_{it}=\lambda W\times \varepsilon_{jt}+v_{it} \end{array}$$

λ is the spatial error coefficient, reflecting the direction and the degree of the influence of the explanatory variable’s bias on local environmental quality, and v*_it_* represents random error term.

### 4.2. Explanation of Variables

Given the accessibility of the hidden economic data in different provinces, the temporal range of data in this study starts from 1998 to 2017. There are 31 provinces in mainland China (excluding Hong Kong, Macao and Taiwan). Tibet Autonomous Region (on the same level with a province) is excluded due to critical data missing. Before Chongqing became a municipality directly under the central government (on the same level with a province) in 1997, it was only a prefecture-level city in Sichuan province. However, some data of Sichuan and Chongqing we use in this study were still being released together until several years later. For the sake of consistency and comparability, the data of Sichuan and Chongqing are merged. Therefore, this study uses the panel data of 29 provinces during 20 years from 1998 to 2017. The variables are illustrated as follows.

Environmental pollution (EP) includes air pollution, water pollution, noise pollution, soil pollution, etc. There is no unified standard for the measurement of environmental pollution in the existing literature. Some studies adopt comprehensive and indirect environmental pollution index such as Environmental Performance Index to measure the level of environmental pollution [12], but most previous studies adopt specific environmental pollution index, such as emissions of carbon dioxide (CO_2_), sulfur dioxide (SO_2_) and nitrous oxide (NO_X_) [53,54]. As for China, some studies choose carbon dioxide or sulfur dioxide as the indicator of environmental pollution [55,56,57], while others choose one or several pollutants of three industrial wastes (waste gas, waste water and solid waste) [1,58,59]. All the indicators above can directly measure the state of environmental pollution in Chinese provinces. This study selects waste gas (including sulfur dioxide) and waste water as the indicators of environmental pollution because industrial pollution is the main cause of environmental pollution in China, and the data of these three industrial wastes are directly from *China Statistical Yearbook* released by China’s National Bureau of Statistics (NBS), which ensures the accuracy of the data. In addition, compared with single indicator like carbon dioxide or sulfur dioxide, multiple indicators can be used to test the robustness of the empirical results. We exclude solid waste because the comprehensive utilization rate of industrial solid wastes improved continually in recent years in China, with the annual discharge amount decreasing from 31.86 million tons in 2000 to 730,000 tons in 2017. The discharge volume of industrial solid waste in many provinces has been less than 10,000 tons since 2008 [60]. These indicate that industrial solid wastes have no longer been the major pollutants in China. For the reasons presented above, this study finally selects per capita industrial waste gas emission (EP1) and per capita industrial waste water discharge (EP2) as proxy variables of environmental pollution.

COR stands for the level of corruption. Corruption is highly invisible, thus making it quite hard to accurately estimate the number of corruptive activities in each region. With an aim to measure the level of corruption in China’s provinces, an objective indicator is required. Common corruption indicators include the Corruption Perception Index (CPI) put forward by Transparency International (TI), the Control of Corruption Index issued by World Bank and the International Country Risk Guide index (ICRG index) proposed by PBS, among which CPI and ICRG index are the most commonly used ones [7,10,12,18,29].

Although the CPI and ICRG index are relatively objective and fair, they only disclose the overall level of corruption of one country (or region) instead of targeting at different regions of one country. Therefore, they are mainly used for empirical analysis at the national level and cannot be applied in this study which aims to analyze the influence of corruption at the provincial level on environmental pollution. Li [61] summarizes the indicators of corruption used in research related to China and divides them into three groups: Perception-based, supply-side and demand-side measures. For perception-based measures of corruption, the only available data at the provincial level are from Asian Barometer Survey (ABS), which is an applied research program designed to assess public opinion on issues such as Asian political values, democracy and governance. However, the data only cover two short periods for China: March–June 2002 and November 2007 to December 2008. In addition, perception-based measures have been criticized for being biased and endogenous [62]. As a result, they are seldom used in the academic research related to China. Supply-side measures can be obtained by examining the company’s public audit reports or accounting statements. In China, researchers use entertainment and travel expenses of companies as a measure of corruption [63] because Chinese managers often submit false or inflated receipts to cover expenses used to bribe government officials and entertain customers and suppliers. The indicator is seldom used in academic research either because the only available data are from the Enterprise Survey conducted jointly by the World Bank and the National Bureau of Statistics, which was only conducted in 2004.

Compared with perception-based and supply-side measures, demand-side measures of corruption are more objective. Fisman and Gatt [64] measure the level of corruption of the states in the USA by using the number of public servants abusing their power. They argue that conviction data are superior to survey-based because they are less subjective, not subject to sampling errors and covers a longer time. Referring to their study, almost all research related to corruption in China adopts the number of duty crime cases (e.g., corruption, bribery, malpractice and infringement) filed for investigation by procuratorial organs, which is disclosed in each province’s work report in the *Procuratorial Yearbook of China*, to indicate the corruption level of each province [1,65,66]. Some scholars question the validity of the indicator. They argue that the number of duty crime cases may not be used to measure corruption but rather the reflection of efficacy of enforcement, unless anti-corruption efforts are equal in all provinces, which is a very implausible assumption [61,62]. Despite the potential shortcomings, considering the availability, objectivity and accuracy of the data, we follow most previous studies related to corruption in China and adopt the number of duty crime cases to measure the corruption level of each province. In order to eliminate the influence of government size and population, we finally select the number of duty crime cases per public servant (COR1) as the proxy variable of corruption. To ensure the robustness of regression result, this study adopts the number of duty crime cases per capita in each province (COR2) as the alternative variable of corruption. Data related to duty crimes in different provinces are obtained from the *Procuratorial Yearbook of China*, with the number of public servants referring to the number of people employed for public management, social security and social organizations by the end of the year in *China Labor Statistical Yearbook*. According to predictions of the above theoretical model, a higher corruption level is accompanied with more severe environmental pollution, so COR1 and COR2 are expected to be positive.

Hidden economy (SE) can be calculated by Equations (6) and (7). W is an N × N symmetric matrix with element w_ij_ and is used to measure the geographical distance or the economic tie between different regions. Diagonal elements w_11_ = … = w_nn_ = 0 imply that the distance between a certain region and itself equals zero. Following Mirshojaeian and Rahbar [67], Maddison [68] and Qian et al. [69], this study adopts three kinds of weight matrix. The first one is geographical distance weight matrix W_D_. If i ≠ j, then w_ij_ = 1/d_ij_; if i = j then w_ij_ = 0, in which d_ij_ is defined as the Euclidean distance between two provincial capitals. Apparently, the closer two provinces are, the bigger geographical distance weight is and the greater interaction they have. The second one is economic distance weight matrix W_E_. We use the reciprocal value of the difference between per capita gross provincial products of two provinces to measure interregional economic weight. If i ≠ j, then w_ij_ = 1/|GDP_i_ − GDP_j_|, where GDP_i_ and GDP_j_ are per capita GDP in province i and j, respectively. The economic distance weight matrix takes differences of economic development in different regions into account. Regions at the same level of economic development have stronger spatial correlation in environmental pollution [68,69]. The third one is mixed weight matrix W_M_. W_M_ = W_D_*W_E_, where W_D_ is geographical distance weight and W_E_ is economic distance weight. It takes the interaction of geographical distance and economic distance on economic activities into consideration.

Z stands for control variables. According to the existing studies on the influencing factors of pollution emission, the following variables are selected to reduce the estimation bias: Economic development, industrial structure, population density, opening degree, energy efficiency and urbanization rate. In order to study the existence of Environmental Kuznets Curve (EKC) between environmental pollution and economic development in Chinese provinces [70,71], the level of economic development (Y) and its quadratic term represented by per capita GDP are introduced to estimate potentially the non-linear relationship, and they are adjusted to real terms at 1998 prices with the per capita GDP deflator index.

The industrial structure (IS) of the economy may affect pollution, and different industrial structures have different influences on environmental pollution [70,71,72]. We measure the industrial structure by the ratio of the added value of the secondary industry in GDP. Due to the high energy consumption and emission of the secondary industry, the higher the ratio of value added by secondary industry in GDP is, the higher the pollution emission will be.

The effect of population density (PD) on environmental pollution is ambiguous [73,74]. On the one hand, the accumulation of population in urban areas causes environmental pollution. On the other hand, high population density makes it possible to use energy more efficiently and intensively. Consequently, the net impact of population density on the environment depends on the relative strength of the two opposite effects above. We measure the population density with per land area at the end of the year.

The effect of opening degree (OPEN) on environmental pollution is ambiguous as well [75]. On the one hand, highly advanced environmental protection technologies can be introduced through foreign trade, which will help reduce pollution. It is defined as the “technical effect” of Grossman and Krueger [76]. On the other hand, as some of the exports are made in China’s energy-intensive and pollution-intensive industries, increased foreign trade may lead to pollution [75]. We measure the opening degree with the value of import and export’ share in GDP.

Energy efficiency (EE), which is represented by energy consumption per real GDP, can reflect the technical level of production, energy input of industrial production and pollution emission [77].

We measure the urbanization rate (URB) with urban population’s share of total population at the end of the year. Many studies show that urbanization is often accompanied by the consumption of large quantities of natural resources and serious pollution emissions [1,57]. Explanations of all the variables are shown in Table 2.

The value form of all variables listed above is adjusted to their actual value in 1998 according to relevant price index. In order to eliminate the influence of different dimensions, all absolute values are transformed into their natural logarithm, and other values remain in their original form. To avoid potential multicollinearity, we calculate the correlation coefficient between corruption and hidden economy before the regression analysis. The correlation coefficient between COR1 and SE is −0.19, and −0.05 for COR2 and SE, which means little correlation between the two variables.

### 4.3. Empirical Results and Analysis

Since SLM model in Equation (9) and SEM model in Equation (10) are both calculated with the global spatial correlation, the spatial lag variables may be endogenous [30]. The common shock of economic behaviors will lead to strong spatial correlation in disturbance terms in the model. If the traditional ordinary least square method (OLS) is used for the estimation, the estimated coefficient will be biased and invalid [78]. Anselin [30] recommends the maximum likelihood estimation (MLE) to estimate the parameters in SEM and SLM, which is widely used in the spatial econometric analysis [79,80,81]. This is because, on one hand, the MLE method can effectively overcome the estimation errors caused by endogeneity in traditional OLS estimation; on the other hand, the real source of spatial features can be identified through the comparison of likelihood values [82]. As a result, the impact of environmental quality of neighboring provinces on that of the province being analyzed can be accurately measured. Referring to the research above, this study adopts the maximum likelihood estimation (MLE) to estimate the spatial regression model. The results of the Hausman test suggest that the fixed effect model is superior to the random effect model. Moreover, the comparison between statistics of LM and LM Robustness Test indicates that LM Lag and LM Lag (Robust) are significant at the 5% level at least, while LM Error and LM Error (Robustness) are non-significant statistically. According to the discrimination criteria for spatial models put forward by Anselin et al. [83], the Spatial Lag Model (SLM) is appropriate. The results are shown in Table 3.

Columns (1) to (6) in Table 3 present the estimation results of the whole sample in 29 provinces concerning the influence of corruption and hidden economy on environmental pollution. Based on the analyses above, it seems that the spatial lag coefficient ρ is significantly positive at the 5% level at least, whether EP1 or EP2 is selected as the indicator of environmental pollution, or whether W_D_, W_E_ or W_M_ is applied, indicating that regional pollutant discharge is characterized by significant overflowing and spatial effects [68,69,83]. In other words, high-polluting regions are adjacent to other high-polluting regions, and so are low-polluting regions.

Indicators of corruption are all significantly positive, suggesting that corruption can increase enterprises’ actual pollutant discharge through weakening the enforcement intensity of environmental regulations or distorting environmental policies, which is consistent with the conclusion of an earlier study [84]. If polluting enterprises bribe officials of environmental protection sectors to weaken the enforcement intensity of environmental regulations, enterprises that used to be forbidden due to low level of production technologies and inconformity to environmental regulations can conduct production. This leads to the increase of gross output and the rise of the average pollution intensity. Through bribery, enterprises that used to apply clean technologies and advanced emission-control devices abandon the application of such devices and switch to polluting technologies which could bring them more profits. Through collusion with officials, these enterprises could partly evade environmental taxes or pay lower taxes. All these will eventually result in increase of discharge of pollutant. A further comparison suggests that the coefficient of corruption is smaller when EP1 is the environmental indicator rather than EP2, which shows that compared with enterprises that discharge industrial waste gas, enterprises discharging industrial waste water are more sensitive to corruption and are more willing to bribe officials of environmental protection sectors in order to weaken the supervision intensity and discharge extra pollutants.

The coefficients of hidden economy are all significantly positive with only marginal difference among equations, indicating that the expansion of the hidden economy’s size could give rise to pollutant discharge. One of the effects of the hidden economy is to evade restrictions of all government regulations and policies including environmental regulations. Compared with the formal economy of the same scale, the hidden economy is bound to discharge more pollutants. Therefore, the expansion of the hidden economy’s size will significantly promote pollutant discharge.

The coefficients of the interaction term of corruption and hidden economy are all significantly positive, with only marginal difference among equations, indicating that corruption could indirectly promote pollutant discharge through lessening the enforcement intensity of environmental regulations and expanding the hidden economy’s size respectively. In other words, the intensification of corruption could increase the operating cost of formal sectors and weaken the supervision intensity on hidden economy. Therefore, there are increasing incentives for enterprises to transfer production activities of formal sectors into the hidden economy, which leads to the expansion of the hidden economy’s size and the increase of the pollutant discharge.

The conclusion of our research accords with reality. In regions with high level of corruption, there are prominent issues of embezzlement and power abuse for personal gain, making explicit and implicit taxes undertaken by enterprises heavier. It is quite difficult to supervise the hidden economy since its production activities are carried out flexibly and invisibly. Moreover, corruption undermines the official’s actual supervision capacity, resulting in the lack of supervision for hidden economic activities. Therefore, enterprises are driven to transfer or directly outsource part of their production activities to hidden economic sectors, which expands the hidden economy’s size and increases the pollutant discharge. In addition, in accordance with the above analysis on the reasons behind the hidden economy, it can be inferred that the severer the corruption is, the higher the percentage that government sectors account for in primary distribution and the lower the resident sectors’ income. The slower the economy grows, the higher the unemployment rate in formal sectors is. Low administrative efficiency usually indicates weak government regulation. The form of individual operation with the purpose of evading taxes and supervision suggests a higher self-employment rate. All these will lead to the increase of the hidden economy’s size which will further boost the pollutant discharge.

### 4.4. Robustness Test

The following robustness tests are conducted to confirm the results. Firstly, in order to test the robustness of the results of empirical analysis for different weight matrixes and pollution indicators, the economic weight matrix and the mixed weight matrix as well as the industrial waste water discharge are also used in addition to the geographical distance weight matrix and the industrial waste gas emission, as shown in Table 3. It can be inferred by the above analysis that the sign of the estimated coefficients of key explanatory variables such as corruption, hidden economy and their interaction term remains the same under the same pollution indicator and different weight matrixes, with only marginal difference in the coefficient estimation. The coefficients of key explanatory variables remain the same under different pollution indicators, indicating the robustness of empirical results. Secondly, apart from selecting COR1 as the measurement of corruption level, COR2 is adopted as substitute variable for corruption to make regression analysis, the test results are as shown in Table 4.

As can be seen from Table 4, if the COR2 is selected as the indicator of corruption, the coefficients of corruption remain positive and significant at the level of 5% at least. The coefficients of hidden economy remain positive and significant at the level of 5% at least. The coefficients of the interaction term are positive and significant at the level of 5% at least. The results above indicate that the estimation results of key explanatory variables in Table 4 are basically consistent with those in Table 3, except for a slight change in significance, which further proves the robustness of the empirical results.

### 4.5. Empirical Results in Different Regions

There are significant differences in the level of economic development and the degree of marketization in the eastern, central and western regions in China, which may affect the influence of corruption and hidden economy on environmental pollution. Therefore, this study divides all provinces into three regions, namely, eastern, central and western regions, and explores the discrepancies concerning the influence of corruption and hidden economy on environmental pollution in different samples. The specific form of spatial econometric model should be determined by combining Hausman test and LM test previous to the empirical analysis. It is shown from the results that the spatial lag model (SLM) with fixed effect is applicable to all the eastern, central and western regions. The results are shown in Table 5, in which each model selects the geographical distance matrix W_D_.

It can be seen from Table 5 that the spatial hysteresis coefficients of models (1) to (6) are significantly positive whether using per capita industrial waste gas emission (EP1) or per capita industrial waste water emission (EP2), indicating that the pollution discharge between the provinces in the eastern, central and western regions is characterized by significant overflowing and spatial effects.

The coefficients of interaction term between corruption and hidden economic in the regression equation of industrial waste gas and industrial waste water in the eastern region are slightly lower than those in the central and western regions, indicating that the promoting effect of the interaction between corruption and hidden economy on pollution discharge in the eastern region is lower than that in the central and western regions, yet with little difference. The reason is that compared with the central and western regions, the overall level of economic development and production technology in the eastern region is more advanced. Correspondingly, the productivity of the hidden economic sector is also higher, and the environmental pollution caused by the same level of corruption and scale of hidden economy is relatively small. Residents in the eastern region have stronger awareness of environmental protection. There are more non-governmental environmental protection organizations. Besides, the pollution emission behavior of hidden economic sectors is better supervised and restricted by the public, leading to less emissions than in the central and western regions under the same level of corruption and scale of hidden economy. Therefore, the interaction between hidden economy and corruption has a lower impact on pollution discharge in the eastern region than in the central and western regions.

### 4.6. Discussion

With different weight matrixes, pollution indicators and measures of corruption, we derived robust empirical results. However, the key explanatory variable in this study, corruption, which is measured by the number of duty crime cases in each province, may be questionable. The indicator may not be used to measure corruption unless anti-corruption efforts are equal in all provinces, which is a very strong and implausible assumption and may affect the validity of the empirical results. However, the indicator is the best one we have been able to obtain so far. It is much better than other indicators used in related research, in terms of the availability, objectivity and accuracy of the data. If perception-based measures at provincial level, such as indicators like Corruption Perception Index (CPI), can be available in the future, empirical analysis should be conducted again to test the robustness of the results.

## 5. Conclusions

Based on China’s provincial panel data from 1998 to 2017, this study examines the influence of the interaction between local government corruption and hidden economy on environmental pollution by adopting the spatial econometrics model. The first main result is that no matter whether it is the industrial waste gas emission or the water discharge that is selected as the pollution indicator and whether the geographical distance weight matrix, economic weight matrix or mixed weight matrix is applied, the coefficients of the hidden economy remain significantly positive. It indicates that the expansion of the hidden economy’s size could give rise to elevated pollutant discharge. The second conclusion is that the coefficients of the interaction term between corruption and hidden economy remain significantly positive, suggesting that corruption could not only directly but also indirectly promote pollutant discharge respectively by reducing the enforcement intensity of environmental regulations and expanding the hidden economy’s size.

The third message of this paper is that here are discrepancies in regard to the influence of the interaction between corruption and hidden economy on environmental pollution in different regions. The promoting effect of the interaction between corruption and hidden economy on pollution discharge in the eastern region is lower than that in the central and western regions.

Corruption forces the transfer of economic activities of formal sectors to hidden sectors, which indirectly increases the pollutant discharge. The production of informal sectors is free from the restrictions of environmental regulations. Hence the expansion of the hidden economy increases the pollutant discharge. The rise of the proportion of corrupt officials reduces the expected penalty cost of production of informal sectors (i.e., weakens the supervision intensity for the hidden economy) and increases the formal sectors’ operating cost. Consequently, there are increasing incentives for enterprises to transfer their production activities into informal sectors, which expands the hidden economy’s size, i.e., corruption can indirectly promote the pollutant discharge through expanding the hidden economy’s size.

Possible policy suggestions include easing the tax burden on enterprises, lowering enterprises’ motivations to transfer production activities into hidden economic sectors, intensifying the fight against hidden economic activities, and establishing a long-term system so that enterprises are unwilling to and incapable to carry out hidden economic production.

## Figures and Tables

**Table 1:** Estimation results of the MIMIC model.

Variables	(1)	(2)	(3)	(4)	(5)	(6)	(7)
TT							0.12 *** (0.06)
IT	2.16 *** (0.22)	2.01 *** (0.11)	1.72 *** (0.15)	1.75 *** (0.18)	1.71 *** (0.13)	1.65 *** (0.17)	
DT	−1.6 *** (0.27)	−1.2 *** (0.11)	−1.9 *** (0.14)	−1.8 *** (0.17)	−1.9 *** (0.13)	−2.1 *** (0.20)	
INC	−0.06 * (0.03)	−0.1 *** (0.01)	−0.1 *** (0.01)	−0.1 *** (0.01)	−0.1 *** (0.01)		
UNE	0.69 *** (0.14)	0.33 *** (0.12)	0.77 *** (0.13)		0.69 *** (0.12)	0.36 ** (0.18)	1.11 *** (0.16)
GR	−2.2 *** (0.36)	−2.3 *** (0.16)					
SFE	−0.09 *** (0.02)		−0.01 (0.02)	−0.01 (0.01)			−0.02 ** (0.01)
RGDP	1	1	1	1	1	1	1
LAB	−1.08 ** (0.53)	−0.71 * (0.37)	−1.23 *** (0.28)	−1.41 *** (0.26)	−1.36 *** (0.28)	−1.08 *** (0.32)	−0.42 *** (0.39)
$\chi^{2}$	16.74 p = 0.02	19.27 p = 0.01	13.40 p = 0.03	1.89 p = 0.58	0.96 p = 0.75	1.49 p = 0.62	0.23 p = 0.81
df (degree of freedom)	6	4	5	3	4	2	2
RMSEA	0.11	0.12	0.12	0.15	0.07	0.02	0.01
CFI	0.83	0.80	0.88	0.86	0.93	1.00	1.00
SRMR	0.14	0.12	0.13	0.08	0.09	0.05	0.01

Note: ***, **, * represents passing the test in a significant level of 1%, 5%, and 10%, respectively.

**Table 2:** Explanation of variables.

Variable	Meaning	Unit
EP1	Per capita industrial waste gas emission	normal cubic meter/person
EP2	Per capita industrial waste water dischare	ton/person
COR1	Number of duty crime cases per public servant	case/ten thousand people
COR2	Number of duty crime cases per capita	case/million people
SE	Share of hidden economy in GDP	%
Y	Per capita gross domestic product	Yuan/person
IS	Added value of second industry’s share of GDP	%
PD	Population at the end of the year per land area	person/suare kilometer
OPEN	Value of import and export’s share of GDP	%
EE	Energy consumption per real GDP	tons of stadard coal/100,000 yuan
URB	Urban population’s share of total pollution at the end of the year	%

**Table 3:** Influence of corruption and hidden economy on environmental pollution.

Explanatory Variables	EP1			EP2		
	(1)	(2)	(3)	(4)	(5)	(6)
COR1	0.16 **	0.16 **	0.14 **	0.26 **	0.30 ***	0.29 ***
	(0.09)	(0.09)	(0.09)	(0.11)	(0.11)	(0.11)
SE	0.04 **	0.04 **	0.04 **	0.05 **	0.05 **	0.05 **
	(0.02)	(0.02)	(0.02)	(0.02)	(0.02)	(0.02)
COR1* SE	0.01 ***	0.01 ***	0.01 ***	0.01 ***	0.01 **	0.01 **
	(0.01)	(0.01)	(0.01)	(0.01)	(0.01)	(0.01)
Y	1.62 ***	1.61 ***	1.65 ***	0.51 *	0.25 **	0.33 **
	(0.24)	(0.22)	(0.23)	(0.16)	(0.16)	(0.16)
Y^2^	−0.04 ***	−0.04 ***	−0.04 ***	−0.03 ***	−0.02 ***	−0.03 ***
	(0.01)	(0.01)	(0.01)	(0.04)	(0.04)	(0.04)
IS	0.01 ***	0.01 ***	0.01 ***	0.01 ***	0.01 **	0.01 ***
	(0.01)	(0.01)	(0.01)	(0.02)	(0.01)	(0.01)
PD	−0.47 ***	−0.37 **	−0.32 **	−0.77 ***	−0.60 ***	−0.65 ***
	(0.15)	(0.14)	(0.14)	(0.18)	(0.18)	(0.18)
OPEN	0.01 **	0.01 **	0.01 ***	0.01 **	0.01 **	0.01 *
	(0.01)	(0.01)	(0.01)	(0.01)	(0.01)	(0.01)
EE	0.73 ***	0.73 ***	0.73 ***	0.87 ***	0.77 ***	0.79 ***
	(0.05)	(0.05)	(0.05)	(0.07)	(0.07)	(0.07)
URB	0.02 ***	0.02 ***	0.02 ***	0.01 **	0.01 *	0.01 *
	(0.01)	(0.01)	(0.01)	(0.01)	(0.01)	(0.01)
ρ	0.28 ***	0.22 ***	0.18 ***	0.59 **	0.16 **	0.04 **
	(0.06)	(0.04)	(0.04)	(0.13)	(0.07)	(0.06)
R^2^	0.86	0.84	0.79	0.88	0.81	0.79
LM Lag	359 ***	15 ***	34.9 ***	5.45 **	4.61 **	3.32 **
LM Lag (Robust)	357 ***	15 ***	34.8 ***	5.51 **	4.56 **	3.28 **
LM Error	1.91	0.05	0.11	0.01	0.05	0.04
LM Error (Robust)	0.17	0.01	0.01	0.07	0.01	0.01
Weight Type	W_D_	W_E_	W_M_	W_D_	W_E_	W_M_

Note: ***, **, * represents passing the test in a significant level of 1%, 5%, and 10%, respectively.

**Table 4:** Robustness test.

Explanatory Variables	EP1			EP2		
	(1)	(2)	(3)	(4)	(5)	(6)
COR2	0.09 ***	0.08 ***	0.07 ***	0.32 **	0.38 ***	0.37 ***
	(0.11)	(0.11)	(0.11)	(0.13)	(0.13)	(0.13)
SE	0.02 **	0.02 **	0.02 **	0.07 ***	0.08 ***	0.08 ***
	(0.02)	(0.02)	(0.02)	(0.03)	(0.03)	(0.03)
COR2*SE	0.01 ***	0.01 **	0.01 **	0.02 ***	0.02 ***	0.02 ***
	(0.01)	(0.01)	(0.01)	(0.01)	(0.01)	(0.01)
Y	1.58 ***	1.58 ***	1.62 ***	0.56 **	0.31 **	0.38 **
	(0.23)	(0.22)	(0.23)	(0.26)	(0.27)	(0.27)
Y^2^	−0.04 ***	−0.04 **	−0.04 **	−0.04 ***	−0.02 **	0.03 **
	(0.01)	(0.01)	(0.01)	(0.01)	(0.01)	(0.01)
IS	0.01 *	0.01 *	0.01 *	0.01 ***	0.01 **	0.01 ***
	(0.01)	(0.01)	(0.01)	(0.01)	(0.01)	(0.01)
PD	−0.49 ***	−0.38 ***	−0.33 **	−0.81 ***	−0.64 ***	−0.69 ***
	(0.14)	(0.13)	(0.13)	(0.17)	(0.17)	(0.17)
OPEN	0.01 ***	0.01 **	0.01 **	0.01 **	0.01 **	0.01 **
	(0.01)	(0.01)	(0.01)	(0.01)	(0.01)	(0.01)
EE	0.71 ***	0.72 ***	0.71 ***	0.85	0.74 ***	0.76 ***
	(0.05)	(0.05)	(0.05)	(0.01)	(0.07)	(0.07)
URB	0.02 ***	0.02 ***	0.02 ***	0.01 **	0.01 *	0.01 *
	(0.01)	(0.01)	(0.01)	(0.01)	(0.01)	(0.01)
ρ	0.29 ***	0.28 ***	0.23 ***	0.67 ***	0.14 **	0.04 *
	(0.06)	(0.04)	(0.04)	(0.13)	(0.07)	(0.06)
R^2^	0.84	0.88	0.85	0.87	0.87	0.83
Weight Type	W_D_	W_E_	W_M_	W_D_	W_E_	W_M_

Note: ***, **, * represents passing the test in a significant level of 1%, 5%, and 10%, respectively.

**Table 5:** Empirical results of different regions.

Explanatory Variables	Eastern		Central		Western	
	EP1	EP2	EP1	EP2	EP1	EP2
	(1)	(2)	(3)	(4)	(5)	(6)
COR1	0.01 *	0.59 ***	0.02 *	0.71 ***	0.03 *	0.68 ***
	(0.14)	(0.16)	(0.15)	(0.16)	(0.17)	(0.18)
SE	0.01 **	0.14 ***	0.05 **	0.21 ***	0.04 **	0.13 ***
	(0.03)	(0.03)	(0.04)	(0.04)	(0.03)	(0.03)
COR1*SE	0.01 **	0.04 ***	0.02 **	0.06 ***	0.02 **	0.05 ***
	(0.01)	(0.01)	(0.01)	(0.01)	(0.01)	(0.01)
Y	1.08 **	0.36 ***	0.32 **	2.44 ***	1.81 ***	0.44 **
	(0.54)	(0.67)	(0.44)	(0.59)	(0.42)	(0.46)
Y^2^	−0.01 **	−0.02 **	−0.04 *	−0.13 ***	−0.05 **	−0.03 **
	(0.02)	(0.03)	(0.02)	(0.03)	(0.02)	(0.02)
IS	0.01	0.01	0.01 ***	0.01 ***	0.01 **	0.01 **
	(0.01)	(0.01)	(0.01)	(0.00)	(0.00)	(0.00)
PD	−0.89 ***	−0.89 ***	−0.44	−1.69 *	−0.61 *	−0.41
	(0.24)	(0.28)	(0.41)	(0.51)	(0.32)	(0.39)
OPEN	0.01 **	0.01 **	0.01 **	0.01 ***	0.01 **	0.01 **
	(0.01)	(0.01)	(0.01)	(0.01)	(0.01)	(0.01)
EE	0.61 ***	0.67 ***	0.18 *	0.36 ***	0.71 ***	0.88 ***
	(0.09)	(0.12)	(0.10)	(0.12)	(0.11)	(0.14)
URB	0.02 ***	0.01	0.02 ***	0.01	0.01	0.01
	(0.01)	(0.01)	(0.01)	(0.01)	(0.01)	(0.01)
ρ	0.01 **	0.36 ***	0.28 ***	0.09 **	0.34 ***	0.56 ***
	(0.08)	(0.12)	(0.08)	(0.12)	(0.08)	(0.15)
R^2^	0.83	0.80	0.83	0.92	0.89	0.84
Weight Type	W_D_	W_D_	W_D_	W_D_	W_D_	W_D_

Note: ***, **, * represents passing the test in a significant level of 1%, 5%, and 10%, respectively.

## Appendix A

**Table A1:** 1998–2017 Provincial hidden economy scale (%) (Part 1).

Province	1998	1999	2000	2001	2002	2003	2004	2005	2006	2007
Beijing	14.01	14.31	15.99	20.13	16.62	13.01	13.93	20.30	19.11	19.68
Tianjin	6.00	11.36	12.50	13.89	14.53	13.88	13.84	13.46	13.20	13.44
Hebei	7.34	8.13	8.99	11.39	13.02	14.10	14.38	14.13	13.71	13.92
Liaoning	10.31	10.82	11.17	9.74	18.92	18.96	19.18	16.59	15.10	12.84
Shanghai	9.71	11.48	11.48	1.75	14.72	14.75	14.08	1.09	13.07	13.12
Jiangsu	9.18	9.96	11.53	13.01	14.84	14.55	13.43	12.11	11.58	11.01
Zhejiang	13.18	14.51	14.76	16.26	17.82	17.48	17.11	15.62	14.58	13.73
Fujian	9.70	10.50	12.47	17.83	19.23	18.51	18.23	18.11	18.00	18.04
Shandong	10.70	10.69	11.28	11.77	12.53	12.24	11.47	11.22	11.12	10.93
Guangdong	8.74	9.20	9.96	11.56	11.83	13.11	10.00	9.18	9.25	9.06
Hainan	17.02	17.13	17.11	17.98	16.28	20.86	17.54	18.39	18.66	18.20
Eastern China Average	10.53	11.65	12.48	13.21	15.48	15.59	14.84	13.66	14.31	14.00
Shanxi	7.27	7.31	7.77	9.02	11.22	9.67	10.13	10.26	10.82	10.80
Jilin	6.36	6.98	7.89	6.93	7.82	9.44	8.87	8.87	8.82	8.35
Heilongjiang	4.19	4.58	6.15	8.70	9.10	7.78	8.42	8.22	8.16	8.01
Anhui	10.95	11.24	11.54	12.80	13.60	13.94	14.28	14.92	14.47	14.21
Jiangxi	11.95	12.80	14.30	16.28	16.44	17.30	17.13	16.42	16.86	16.18
Henan	10.23	10.55	10.75	11.68	12.05	12.63	13.61	13.67	13.91	13.69
Hubei	12.86	13.68	14.81	16.84	17.87	17.72	17.43	17.86	17.41	17.35
Hunan	9.19	9.44	9.23	10.08	10.09	11.22	10.89	10.57	10.65	10.70
Central China Average	9.12	9.57	10.31	11.54	12.27	12.47	12.59	12.60	12.64	12.41
Neimenggu	13.17	13.45	14.08	15.46	17.09	19.07	19.88	18.62	17.85	17.41
Guangxi	6.05	6.41	6.31	7.05	7.21	6.70	7.80	7.82	7.79	7.14
Sichuan and Chongqing	7.78	7.83	8.09	8.91	9.24	8.58	8.93	9.29	9.08	8.60
Guizhou	15.47	16.85	16.20	17.06	17.45	17.31	17.37	17.74	17.37	17.13
Yunnan	5.05	6.02	6.28	7.57	8.83	7.80	9.20	8.91	9.08	9.06
Shaanxi	16.99	14.86	15.26	18.02	17.86	20.97	21.08	22.88	22.34	22.28
Gansu	16.56	14.69	14.38	15.00	16.78	22.13	17.38	16.58	18.28	16.90
Qinghai	15.79	16.19	15.43	21.34	21.67	20.85	22.69	22.68	22.49	22.29
Ningxia	15.95	15.61	16.01	15.65	15.15	10.53	15.31	15.34	14.55	14.25
Xinjiang	9.12	8.92	9.05	8.85	8.86	6.93	8.20	9.07	9.00	9.19
Western China Average	12.19	12.08	12.11	13.49	14.01	14.09	14.78	14.89	14.78	14.43
National Average	10.72	11.22	11.75	12.85	14.09	14.21	14.20	13.79	14.01	13.71

**Table A2:** 1998–2017 provincial hidden economy scale (%) (Part 2).

Province	2008	2009	2010	2011	2012	2013	2014	2015	2016	2017
Beijing	20.58	16.70	15.71	17.11	15.81	15.30	13.09	12.42	12.27	11.24
Tianjin	13.52	13.63	13.69	13.89	13.82	14.06	13.80	13.70	13.28	13.25
Hebei	14.50	14.05	14.47	14.02	13.97	14.15	13.78	13.60	13.49	13.77
Liaoning	11.77	11.55	10.99	11.25	10.96	10.38	9.88	9.16	10.92	10.94
Shanghai	12.96	12.90	13.08	10.86	9.39	11.80	12.19	12.69	11.62	11.53
Jiangsu	10.76	10.59	10.13	10.28	10.00	9.21	9.05	8.79	7.86	7.80
Zhejiang	14.64	13.73	13.35	13.20	12.71	12.28	11.10	9.85	10.27	9.14
Fujian	17.69	17.46	16.88	16.61	16.26	16.25	15.52	15.67	15.54	15.75
Shandong	12.52	11.46	11.33	11.29	11.26	10.96	10.93	10.76	10.68	10.52
Guangdong	9.23	9.32	9.02	9.05	9.06	8.83	8.34	7.97	7.31	7.65
Hainan	20.23	19.32	18.09	11.95	13.41	13.96	14.86	14.85	14.46	13.91
Eastern China Average	14.40	13.70	13.34	12.68	12.42	12.47	12.05	11.77	11.61	11.41
Shanxi	11.15	12.94	11.85	11.56	11.16	10.60	11.19	11.21	10.90	11.04
Jilin	8.45	8.31	7.80	7.60	7.47	7.45	6.69	6.65	6.50	6.50
Heilongjiang	7.88	7.94	7.86	7.58	7.60	8.13	8.35	8.56	7.87	7.98
Anhui	13.92	14.01	13.25	13.56	13.42	12.61	11.82	11.44	11.19	10.22
Jiangxi	16.25	16.42	15.77	14.12	14.58	15.67	15.99	16.14	15.97	15.23
Henan	13.63	13.89	13.41	13.30	12.33	12.81	12.19	12.07	11.85	11.17
Hubei	17.21	17.02	16.82	16.38	15.22	13.51	11.39	9.44	8.13	8.90
Hunan	10.40	10.26	10.23	10.20	10.30	10.28	10.05	9.53	10.15	9.52
Central China Average	12.36	12.60	12.12	11.79	11.51	11.38	10.96	10.63	10.32	10.07
Neimenggu	17.59	17.10	16.92	16.47	15.91	15.14	14.57	15.01	15.13	15.04
Guangxi	7.04	7.05	6.92	6.53	6.40	6.22	5.89	5.35	5.15	3.97
Sichuan and Chongqing	9.24	8.80	8.65	8.66	8.34	8.50	8.36	7.91	7.64	7.10
Guizhou	17.01	16.46	16.01	15.95	14.79	14.59	14.53	14.10	13.00	12.80
Yunnan	9.14	9.25	9.29	8.94	8.91	8.75	8.48	8.35	7.34	6.56
Shaanxi	21.86	22.16	21.88	20.48	17.75	17.95	18.17	17.99	17.48	17.64
Gansu	16.43	16.42	16.17	15.66	13.79	12.04	11.42	11.16	10.88	13.34
Qinghai	22.55	22.97	22.92	22.91	20.85	21.06	20.16	20.27	18.77	18.89
Ningxia	14.58	14.66	14.50	14.74	14.14	14.02	13.47	12.95	11.90	12.08
Xinjiang	8.90	9.24	8.03	8.42	8.72	8.81	8.24	7.33	6.54	6.65
Western China Average	14.43	14.41	14.13	13.88	12.96	12.71	12.33	12.04	11.38	11.41
National Average	13.85	13.64	13.28	12.85	12.36	12.25	11.84	11.55	11.18	11.04

## References

[1] Chen H., Yu H., Li J., Song X. (2018). The impact of environmental regulation, shadow economy, and corruption on environmental quality: Theory and empirical evidence from China. J. Clean. Product..

[2] Huang P.C. (2009). China’s neglected informal economy: Reality and theory. Mod. China.

[3] Feige E.L. (1994). The underground economy and the currency enigma. Public Finance.

[4] Blackman A. (2000). Informal sector pollution control: What policy options do we have?. World Dev..

[5] Desai U. (1998). Ecological Policy and Politics in Developing Countries: Economic Growth, Democracy, and Environment.

[6] Pellegrini L., Gerlagh R. (2006). Corruption and environmental policies: What are the implications for the enlarged EU?. Environ. Policy Gov..

[7] Ivanova K. (2011). Corruption and air pollution in Europe. Q. J. Econ..

[8] Hubbard T.N. (1998). An empirical examination of moral hazard in the vehicle inspection market. Rand J. Econ..

[9] Oliva P. (2015). Environmental regulations and corruption: Automobile emissions in Mexico City. J. Polit. Econ..

[10] Zhang Y.J., Jin Y.L., Chevallier J., Shen B. (2016). The effect of corruption on carbon dioxide emissions in APEC countries: A panel quantile regression analysis. Technol. Forecast. Soc. Chang..

[11] Lisciandra M., Migliardo C. (2017). An empirical study of the impact of corruption on environmental performance: Evidence from panel data. Environ. Res. Econ..

[12] Chang C.P., Hao Y. (2017). Environmental performance, corruption and economic growth: Global evidence using a new data set. Appl. Econ..

[13] Lapatinas A., Litina A., Sartzetakis E.S. (2018). Environmental projects in the presence of corruption. Int. Tax Public Finance.

[14] Wang Z., Danish, Zhang B., Bo W. (2018). The moderating role of corruption between economic growth and CO_2_ emissions: Evidence from BRICS economies. Energy.

[15] Dincer O.C., Fredriksson P.G. (2018). Corruption and environmental regulatory policy in the United States: Does trust matter?. Res. Energy Econ..

[16] Arminen H., Menegaki A.N. (2019). Corruption, climate and the energy-environment-growth nexus. Energy Econ..

[17] Dirienzo C.E., Das J. (2019). Women in government, environment, and corruption. Environ. Dev..

[18] Sinha A., Gupta M., Shahbaz M., Sengupta T. (2019). Impact of corruption in public sector on environmental quality: Implications for sustainability in BRICS and next 11 countries. J. Clean. Prod...

[19] Balsalobre-Lorente D., Shahbaz M., Jabbour C.J.C., Driha O.M. (2019). The role of energy innovation and corruption in carbon emissions: Evidence based on the EKC hypothesis. Energy Environ. Strateg. Era Glob..

[20] Huynh C.M., Hoang H.H. (2019). Foreign direct investment and air pollution in Asian countries: Does institutional quality matter?. Appl. Econ. Lett..

[21] Chaudhuri S., Mukhopadhyay U. (2006). Pollution and informal sector: A theoretical analysis. J. Econ. Integr..

[22] Baksi S., Bose P. (2010). Environmental regulation in the presence of an informal sector. Am. J. Cardiol..

[23] Elgin C., Mazhar U. (2013). Environmental regulation, pollution and the informal economy. SBP Res. Bull..

[24] Abid M. (2015). The close relationship between informal economic growth and carbon emissions in Tunisia since 1980: The (ir)relevance of structural breaks. Sustain. Cities Soc..

[25] Imamoglu H. (2018). Is the informal economic activity a determinant of environmental quality?. Environ. Sci. Pollut. Res..

[26] Canh N.P., Thanh S.D., Schinckus C., Bensemann J., Thanh L.T. (2019). Global emissions: A new contribution from the shadow economy. Int. J. Energy Econ. Policy.

[27] Zhou Z. (2019). The underground economy and carbon dioxide (CO_2_) emissions in China. Sustainability.

[28] Elgin C., Oztunali O. (2014). Pollution and informal economy. Econ. Syst..

[29] Biswas A.K., Farzanegan M.R., Thum M. (2012). Pollution, shadow economy and corruption: Theory and evidence. Ecol. Econ..

[30] Anselin L. (1988). Spatial Econometrics: Methods and Models.

[31] Cantekin K., Elgin C. (2017). Extent and growth effects of informality in Turkey: Evidence from a firm-level survey. Singap. Econ. Rev..

[32] Medvedev D., Oviedo A.M. (2016). Informality and profitability: Evidence from a new firm survey in Ecuador. J. Dev. Stud..

[33] Danopoulos C.P., Znidaric B. (2007). Informal economy, tax evasion, and poverty in a democratic setting: Greece. Mediterr. Q..

[34] Elgin C., Erturk F. (2019). Informal economies around the world: Measures, determinants and consequences. Eurasian Econ. Rev..

[35] Tanzi V. (1983). The underground economy in the United States: Annual estimates, 1930–1980. IMF Staff Pap..

[36] Orsi R., Raggi D., Turino F. (2014). Size, trend, and policy implications of the underground economy. Rev. Econ. Dyn..

[37] Bajada C., Schneider F. (2005). The shadow economies of the Asia-Pacific. Pac. Econ. Rev..

[38] Dell’anno R., Solomon O.H. (2008). Shadow economy and unemployment rate in USA: Is there a structural relationship? An empirical analysis. Appl. Econ..

[39] Popescu G., Davidescu A., Huidumac C. (2018). Researching the main causes of the Romanian shadow economy at the micro and macro levels: Implications for sustainable development. Sustainability.

[40] Giles D.E.A. (1999). Modelling the hidden economy and the tax-gap in New Zealand. Empir. Econ..

[41] Alañón A., Gómez-Antonio M. (2005). Estimating the size of the shadow economy in Spain: A structural model with latent variables. Appl. Econ..

[42] Thomas J.J. (1992). Informal Economic Activity.

[43] Schneider F., Buehn A., Montenegro C.E. (2010). Shadow economies all over the world: New estimates for 162 countries from 1999 to 2007. World Bank Policy Research Working Paper.

[44] Schneider F.G. (2005). Shadow economies around the world: What do we know?. Eur. J. Polit. Econ..

[45] Schneider F., Dell’anno R. (2003). The shadow economy of Italy and other OECD countries: What do we know?. J. Publ. Finance Public Choice.

[46] Schneider F. (1986). Estimating the size of the Danish shadow economy using the currency demand approach: An attempt. Scand. J. Econ..

[47] Schneider F., Enste D.H. (2000). Shadow economies: Size, causes, and consequences. J. Econ. Lit..

[48] Saafi S., Farhat A. (2015). Is there a causal relationship between unemployment and informal economy in Tunisia: Evidence from linear and non-linear Granger causality. Econ. Bull..

[49] Ihrig J., Moe K.S. (2004). Lurking in the shadows: The informal sector and government policy. J. Dev. Econ..

[50] Bordignon M., Zanardi A. (1997). Tax evasion in Italy. Giornale Degli Economisti e Annali di Economia.

[51] Jin-Chang L., Ai-Ting X. (2005). Research on estimation method of non-observed economy. Stat. Res..

[52] Tobler W. (1970). A computer movie simulating urban growth in the Detroit region. Econ. Geogr..

[53] Danaeifar I. (2014). The estimation parameters of Kuznets spatial environmental curve in European countries (a case study of CO_2_ and PM10 and incidence of tuberculosis and life expectancy at birth). Eur. Online J. Nat. Soc. Sci. Proc..

[54] Sinha A., Bhatt M. (2017). Environmental kuznets curve for CO_2_ and NOx emissions: A case study of India. Eur. J. Sustain. Dev..

[55] Ge X., Zhou Z., Zhou Y., Ye X., Liu S. (2018). A spatial panel data analysis of economic growth, urbanization, and NOx emissions in China. Int. J. Environ. Res Public Health.

[56] Dong K., Hochman G., Kong X., Sun R., Wang Z. (2019). Spatial econometric analysis of China’s PM10 pollution and its influential factors: Evidence from the provincial level. Ecol. Indic..

[57] Wang Y., Han R., Kubota J. (2016). Is there an environmental Kuznets curve for SO_2_ emissions? A semi-parametric panel data analysis for China. Renew. Sustain. Energy Rev..

[58] Tao S., Zheng T., Lianjun T. (2008). An empirical test of the environmental Kuznets curve in China: A panel cointegration approach. China Econ. Rev..

[59] He J., Wang H. (2012). Economic structure, development policy and environmental quality: An empirical analysis of environmental Kuznets curves with Chinese municipal data. Ecol. Econ..

[60] National Bureau of Statistics of China (2018). China Statistical Yearbook 2018.

[61] Li X. (2016). Measuring local corruption in China: A cautionary tale. J. Chin. Polit. Sci..

[62] Treisman D. (2007). What have we learned about the causes of corruption from ten years of cross-national empirical research?. Annu. Rev. Polit. Sci..

[63] Wang Y. (2014). Institutions and bribery in an authoritarian state. Stud. Comp. Int. Dev..

[64] Fisman R., Gatti R. (2002). Decentralization and corruption: Evidence across countries. J. Public Econ..

[65] Cole M.A., Elliott R.J., Zhang J. (2009). Corruption, governance and FDI location in China: A province-level analysis. J. Dev. Stud..

[66] Xu X., Li Y., Liu X., Gan W. (2017). Does religion matter to corruption? Evidence from China. China Econ. Rev..

[67] Mirshojaeian H.H., Rahbar F. (2011). Spatial environmental Kuznets curve for asian countries: Study of CO_2_ and PM10. J. Environ. Stud..

[68] Maddison D. (2007). Modelling sulphur emissions in Europe: A spatial econometric approach. Oxf. Econ. Pap..

[69] Qian L., Song J., Wang E., Hao H., Zhang J., Wang Y. (2014). Economic growth and pollutant emissions in China: A spatial econometric analysis. Stoch. Environ. Res. Risk Assess..

[70] Yin J., Zheng M., Chen J. (2015). The effects of environmental regulation and technical progress on CO_2_ Kuznets curve: An evidence from China. Energy Policy.

[71] Wang Q., Zeng Y.-E., Wu B.-W. (2016). Exploring the relationship between urbanization, energy consumption, and CO_2_ emissions in different provinces of China. Renew. Sustain. Energy Rev..

[72] Wang P., Wu W., Zhu B., Wei Y. (2013). Examining the impact factors of energy-related CO_2_ emissions using the STIRPAT model in Guangdong Province, China. Appl. Energy.

[73] Lee C.C., Chiu Y.B., Sun C.H. (2009). Does one size fit all? A reexamination of the environmental Kuznets curve using the dynamic panel data approach. Rev. Agric. Econ..

[74] Yu H., Chen H., Zhang Q. (2016). Will income inequality affect environmental quality? Analysis based on China’s provincial panel data. Ecol. Indic..

[75] Du L., Chu W., Cai S. (2012). Economic development and carbon dioxide emissions in China: Provincial panel data analysis. China Econ. Rev..

[76] Grossman G.M., Krueger A.B. (1991). Environmental impacts of a North American free trade agreement. Natl. Bur. Econ. Res..

[77] Sun W., Chen Z., Wang D. (2019). Can land marketization help reduce industrial pollution?. Int. J. Environ. Res. Public Health.

[78] Podojil S., Jacob M.C., Tuccillo C., Maier D. (2010). FDI in Chinese cities: Spillovers and impact on growth. World Economy.

[79] Rey S.J., Montouri B.D. (1999). US regional income convergence: A spatial econometric perspective. Reg. Stud..

[80] Elhorst J.P. (2005). Unconditional maximum likelihood estimation of linear and log-linear dynamic models for spatial panels. Geogr. Anal..

[81] Lacombe D.J. (2004). Does econometric methodology matter? An analysis of public policy using spatial econometric techniques. Geogr. Anal..

[82] Blonigen B.A., Davies R.B., Waddell G.R., Naughton H.T. (2007). FDI in space: Spatial autoregressive relationships in foreign direct investment. Eur. Econ. Rev..

[83] Anselin L., Bera A.K., Florax R., Yoon M.J. (1996). Simple diagnostic tests for spatial dependence. Region. Sci. Urban Econ..

[84] Cole M.A. (2007). Corruption, income and the environment: An empirical analysis. Ecolog. Econ..

